# Model framework for governance of genomic research and biobanking in Africa – a content description

**DOI:** 10.12688/aasopenres.12844.2

**Published:** 2018-12-12

**Authors:** Aminu Yakubu, Paulina Tindana, Alice Matimba, Katherine Littler, Nchangwi Syntia Munung, Ebony Madden, Ciara Staunton, Jantina De Vries

**Affiliations:** 1 Department of Health Planning, Research & Statistics, Federal Ministry of Health, Abuja, Nigeria; 2 Navrongo Health Research Centre, Ghana Health Service, Navrongo, Ghana; 3 Wellcome Genome Campus Advanced Courses and Scientific Conferences, Cambridge, UK; 4 Global Policy, Wellcome Trust, London, UK; 5 Department of Medicine, University of Cape Town, Cape Town, South Africa; 6 Division of Genomic Medicine, National Human Genome Research Institute, National Institutes of Health, Rockville, MD, USA; 7 Law Department, Middlesex University, London, UK

**Keywords:** genomics research, biobanking, governance

## Abstract

Genomic research and biobanking are expanding globally, with a promise to fast-track the research needed to improve approaches to disease treatment and prevention through scientific collaborations such as the Human Heredity and Health in Africa (H3Africa) initiative. Integral to this type of research is the availability of samples and data for research. The need for broad access brings along a host of ethical concerns, including those related to privacy and confidentiality, as well as fairness and equity in access and capacity to utilise these samples between scientists from the high income and low income countries. Addressing these concerns while promoting genomic research, especially in Africa, requires the implementation of a sound governance framework. In this paper, we describe the contents of a Framework for Best Practice for Genomics Research and biobanking in Africa that was developed, under the auspices of the H3Africa initiative. This framework is broad enough to be used and adapted by African countries to facilitate the development of country-specific guidelines and to help improve the conduct and governance of genomics research.

## Introduction

The complete mapping of the human genome ^1^ brought along with it possibilities to better understand human health and its determinants which would help improve the way diseases and health conditions are managed. Since then, the number of genomic research studies and the need for biobanking has been growing globally ^2– 4^, with a steady increase in the number of such studies being conducted on the African continent ^5^. Further scaling up genomics research with samples and data from diverse African populations ^6^ has great value considering that only a small portion of human diversity is found outside Africa ^7^. Towards addressing this gap, a $76 million initiative referred to as the Human Heredity and Health in Africa Initiative (H3Africa) was funded jointly by the National Institutes of Health in the US and Wellcome Trust in the UK ^3^. In order to fast-track realization of the benefits inherent in genomics research, the global community agreed to principles of ‘open science’, which promotes the value of sharing and reuse of data and samples as a critical component of the contemporary scientific landscape ^8– 10^. Although this has the strong potential to facilitate scientific discovery, it also raises a number of ethical concerns which includes appropriate model of consent that will allow for such sharing of data and samples while upholding participant autonomy ^11– 14^; issues of withdrawal of consent ^15, 16^; ownership of samples and data ^17– 20^; privacy and confidentiality ^15, 21– 23^; and benefit sharing ^24– 26^. Such issues are not peculiar to African countries, but require a different lens in further elucidating the contextual concerns in African settings. Furthermore, there are concerns about trust ^27^ and fairness in research collaborations ^10, 28^, increased vulnerability of research participants due to lower socio-economic levels, a history of exploitation of local populations and researchers ^29^, and cultural issues that all must be considered in the governance of genomics research and biobanking in Africa.

In addition to these ethical concerns, regulatory frameworks for health research in Africa are either non-existent, or where they exist, do not respond to the specific concerns raised by genomics research and biobanking ^10, 23, 30^. Towards addressing this gap, the H3Africa Initiative considered it expedient to develop an ethics framework describing best practice for genomics research and biobanking. Described in terms of core principles and elements, the Framework considered African political history of exploitations from the West and accounts of ethical concerns in conducting genomics research and biobanking ^30– 33^ to inform the choice of these core principles and elements. The phenomenon of “parachute research” – where fully equipped research teams from other countries arrive at the site where research is needed, conduct their research independently of others, and then leave, has been long cited as a challenge for genuine collaborative research in Africa as well as other developing countries in the world. That such practices are not part of the past was highlighted during the recent Ebola epidemic in some West African countries where international researchers are said to have carted specimens away from the affected countries without any form of oversight or recourse to local regulations or regulators ^29^. A permutation of these practices is where African researchers are reduced to the role of data and sample collectors, without genuine attempts to ensure their involvement beyond such functions. One way to remedy such practices is by fostering sustainable capacity building for African intellectual leadership in the conceptualization, design, implementation and reporting of locally appropriate studies ^30, 34, 35^ – this is one of the core elements of the framework described here. It is also one of the main goals of the H3Africa initiative including support for equipment and infrastructure to enable researchers to develop biobanks and conduct large-scale genomics studies.

The aim of this framework is to guide governance, address sample and data sharing concerns, as well as to serve as a resource for countries to develop their local regulations. The process of engagement that led to the development of this framework is reported in another manuscrip. ^36^. This paper highlights the key contents of this framework and plans for its implementation across the African continent.

## An overarching philosophical basis

In developing this ethics framework, it was important for key stakeholders to identify the overarching philosophical basis that should guide the development of ethical best practice for genomic research and biobanking. The issues of unfair research collaboration, lack of trust in research collaborations with scientists from the “West” and the related skepticisms among experts and community representatives ^30, 37– 39^ were key considerations. These remain genuine concerns that need to be addressed to promote inclusion of Africans in genomics research as the field progresses and the world benefits from its outcomes ^6^. To ensure that African patients and researchers partake in genomic research to optimal benefit, we thought it important to adopt a normative basis from African cultural philosophy. “Ubuntu” is a largely South African worldview that focuses on the interrelatedness of humans in their quest for mutual co-existence and is one such African philosophy that was initially selected as a foundation for the framework ^40, 41^. There is strong ground to suggest the importance of principles of solidarity as articulated in the Ubuntu philosophy as a universal moral theory for sub Saharan Africa ^42, 43^. However, given its strong historical links to an era of struggle in apartheid South Africa and several accounts limiting it as a southern African philosophy ^44, 45^, its acceptance during our consultations as a philosophical basis for the framework met with some resistance from stakeholders in other parts of Africa. Instead, upon consultation we agreed that the framework should adopt a more generic communal or solidarity-based worldview. Such a worldview recognises that individuals are shaped by their relations to people around them and emphasizes respectful and harmonious relationships between them. In the African research context, we agreed that a communitarian perspective would place central importance on reciprocity, consultation and accountability as key ethical values.

## Core Principles and Elements

The framework proposes a set of four core principles that ought to underpin guidance for genomic research and biobanking initiatives in Africa. Whilst these principles are not new, they need to keep being emphasized to address particular concerns of African communities and scientists regarding trust and unfairness in research collaborations. A number of these principles have been echoed in a parallel initiative, the San Code of Ethics, which seeks to define how researchers ought to conduct their work when dealing with the San population in South Africa ^46, 47^, The four core principles of our framework emphasize the need for research to: a) be sensitive and respectful of African values and cultures; b) be designed primarily to benefit the African people, while acknowledged it may equally benefit the global population; c) ensure genuine and active intellectual participation of African investigators and other stakeholders in research and in dissemination of findings; and d) promote relationships characterised by respect, fairness, equity and reciprocity. As core principles, these aspects are non-negotiable and should be incorporated in the design and conduct of genomics research and biobanking initiatives in Africa.

## Key elements of the Framework for Best Practice

In addition to these core principles, the Framework also describes ten key areas that need to be addressed in order to ensure that the core principles elucidated in the framework are realised. They include: African intellectual leadership; Consent; Community engagement; Ethics review; Avoidance of group harm and stigma; Benefit sharing; Capacity building; International collaboration and export of samples; Feedback of individual genetic findings; and Good governance. These core elements are not mutually exclusive. For instance, high-level capacity building is key to ensuring African intellectual leadership. These elements were informed by a review of guidelines documents and other similar texts from institutions in the global North such as the Global Alliance for Genomics and Health ^48^ and the Organization for Economic Cooperation and Development ^49^ among others, and adapted to African experiences and context, thus ensuring alignment with global best practices.

## Highlights of the key elements of the framework

The gap in research investments between the north and south has been reported previously and considered unacceptable ^50^. The persistence of this gap, more than two decades after it was initially described could strongly mitigate the potential of genomics research to provide novel insights into health disparities, foster better understanding of human biology, support improvement in individual clinical care and informing genetic diagnosis among others ^6^. The underrepresentation of scientists from low and middle income countries (LMICs) ^51^ and African countries in particular ^52^ at all levels of research is one key area of intervention, which when addressed could significantly reduce this gap. Thus, central to this framework is the requirement for all genomics research collaborations in Africa to ensure **African intellectual leadership** – that scientists who are Africans and/or based in African institutions should be engaged and supported to assume leadership roles in the design and implementation of both primary and secondary studies emergent therefrom with the H3Africa project being a good example ^10^. This would further promote an earlier call that “developing countries must have the capacity to investigate their own health priorities” ^53^, as well as the needed diversity to fully harness the potentials of genomics research in improving global health ^10^. We do however recognise that for sustainability, this should be supported by more deliberate efforts by country governments to strengthen their health research systems.

Because sample and data sharing are essential to genomics research and biobanking, it is important that the **consent process** allows sharing and re-use whilst still respecting participant choice. While there are several models of informed consent that may support genomics research ^54^, our framework promotes the use of broad consent. It is important to differentiate this from blanket consent, which is consent for sharing and use of data and specimen without any restrictions. Broad consent as understood in the framework allows for “ *use of samples and/or data for unspecified future studies, but with conditions. These conditions can involve, for instance a restriction on the types of studies or diseases that samples/data can be used for; a specified oversight and approval process for future use; ongoing consultation with sample donors about future use, if possible; and a process allowing participants to withdraw samples or data from the storage facility that holds them*”. Although debates about appropriate consent models for genomics and biobanking continue, there is growing consensus that broad consent may be the ‘best compromise’ consent model ^55^. In their critical presentation of the outcomes of a workshop which aimed to identify the appropriate consent model for collection of biospecimen for use in future research, Grady *et al.* (2015) submitted that broad consent was considered “ethically appropriate, and preferable to lack of consent for the majority of biospecimen collection for future research uses” ^54^. Furthermore, Tindana and De Vries (2016) in their paper on the perspectives of broad consent for genomics research in LMICs concluded that there are no *a priori* reasons against the use of broad consent for genomics research in Africa ^56^. Empirical research conducted since then suggests that participants may also be supportive of broad consent if done sensitively and respectfully ^57– 59^. The proposed framework for acceptable broad consent includes initial consent, oversight of future research projects, and, when feasible, mechanisms for maintaining contact and communication with specimen donors. There is, however, an agreement among most proponents of broad consent that for optimal participant protection, whenever broad consent is used in genomics research to allow for future research use, it needs to be accompanied with a mechanism that promotes accountability and equity ^60^ in sharing specimen and/or data with other researchers while also ensuring that participants’ choice are respected ^54, 56^.

The Framework considers that genuine **community engagement** (CE) is a key component of ensuring best practice in genomics research and biobanking in Africa, not in the least because it promotes respect for community values and perspectives and maximizes the social value of research ^61^. Community engagement is one way to ensure that research conduct is aligned with a communitarian worldview. Furthermore, genuine community engagement is proposed as a condition for the use of broad consent ^54, 56^. To be clear, community engagement as put forward in the framework goes beyond tokenistic engagement which would involve a once-off meeting with some community members and rather denotes substantive engagement with various members of communities over time. The research team should seek to engage relevant communities at whom a particular project is targeted along the entire spectrum of research activities, from initial planning phases and data collection, to include the return of general study findings when projects end and policy translations where applicable. While there are several accounts of what it means to have a genuine community engagement ^62, 63^, to be considered meaningful, researchers should demonstrate that proposed CE initiatives are built on some of the known guiding principles or values of CE; adapting some of the related strategies as applicable ^64, 65^.

The framework recommends that all primary genomic research and biobanking studies must be reviewed by a competent research **ethics review** committee based in the country where samples are collected or stored. Research ethics review fulfils an important role in promoting ethical best practice and is key to the protection of research participants Such a role is very important in the African research that takes place in the context of a high burden of disease, poor access to basic necessities and healthcare, low average income and literacy levels as well as unfamiliarity of most of the people with biomedical research generally and genomic research specifically. A particular challenge is the limited capacity of research ethics committees in Africa to review genomics research and biobanking projects ^66, 67^. Using a matrix that maps the various elements of this framework against important issues that ethics committees are recommended to consider, Table 1 is proposed as a practical tool for ethics committees to provide oversight for good governance in genomics research and biobanking.

**Table 1.** Checklist for Ethics Committee Review of Governance Arrangements in Proposed Genomics Research Studies.

Element	Explanation	Criteria
African intellectual leadership	The substantive contribution of scientists based at African institutions	Intellectual leadership or co-leadership of scientists at African institutions
Broad consent	Consent for the use of samples and/or data for unspecified future studies subject to conditions	Must be fully informed and voluntary Participants must be informed about the manner and extent to which they may withdraw from the study Must be supported by community engagement Must be supported by a good governance framework Must be subject to ethical review
Community engagement (CE)	The process of informing, consulting and actively involving relevant communities that have a legitimate interest in the research process	Must be an integral part of each research project Goals and process of CE must be clearly defined, planned and designed collaboratively This must be done at the start of the research CE must be evaluated
Ethics review	Ethics review promotes ethical conduct of research while providing assurances to the public that their welfare is being well taken care of as they contribute to knowledge and development	Every genomic research and biobanking study must be subject to ethics review Re-use of samples must be subject to review by a designated committee The use of samples must be subject to a sample access committee The use of data must be subject to data access committee review
Avoiding group harm or stigma	The reporting of genomic research results has the potential to aggravate existing stigma or marginalization, or punishment	Donors and research ethics comittees must be told about any risk of group harm or stigma with the use or re-use of samples Stigma related concerns about the sharing of genomic samples and data should be subject to stakeholder engagement Where there are stigma related concerns, individuals from the countries and/or institutions where obtained should be considered Descriptors that may be perceived to be stigmatizing or prejudicial must be avoided
Benefit sharing	Benefit sharing regulates that benefits and burdens are distributed fairly and it is therefore key to ensuring that research collaboration is fair	Genomics research may likely yield intangible benefits like knowledge generation and capacity building, some of which may only translate into tangible benefits in future generations. General study results can count as one study benefit and should be fed back to communities in which research is conducted. This is essential to maintaining trust and can be incorporated in community engagement activities. If there is a realistic expectation of tangible benefit to a group, a benefit sharing plan must be agreed to after stakeholder engagement Consideration must be given to how genomic and biobanking research may confer benefits on participants
Capacity building	Genomic research and biobanking conducted in Africa should lead to substantive building of research capacity, including both human resources and research infrastructure	A capacity building plan must be included as part of each research project This must include infrastructure, personnel and administrative capacity building
International collaboration and export of samples	International collaboration and export of samples should promote the goals of reducing global health inequality and exploitation and strengthening the research system in the country where the samples were collected	Export of samples must be subject to ethics review Exportation of samples and collaboration must help build local capacity Exportation of samples must be indicated in the consent documents Exportation of samples must be subject to materials transfer agreements
Feedback of individual genetic research results	The feedback of findings in the African context considering difficulties of validating research findings in a diagnostic facility, the absence of healthcare workers trained in genetics that could provide feedback, and limited validation of genomic research findings in African populations	There is a need for wide stakeholder engagement to determine when feedback of findings should be provided and a plan on how this would be applied.
Good governance	Good governance helps build and maintain public trust and ensure transparency of genomic and biobanking research	Governance framework must include oversight on the use and reuse of samples There must be compliance with local, national and international guidelines and regulations Entities controlling access to samples must be comprised of members primarily residing on the African continent

The **avoidance of group harm or stigma** is considered important particularly in the African research context where researchers may work with members of many different population groups, each characterised by their own language, culture and belief systems, some of which may be marginalised or discriminated against. Research may also involve groups of people suffering from stigmatised conditions or outlawed or stigmatising behaviours, phenotypes or lifestyles. In such a context, the reporting of genomic research results could aggravate existing stigma or marginalisation. An example is the way in which genomic research on the San included findings that were considered potentially stigmatising ^46, 47^. In this example, Namibian San leaders were approached for participation in genomics research ^68^ without involvement of San political leadership or individuals with experience in science who could have properly explained the research project and who could have helped the research team in designing more appropriate consent processes. Whilst presented internationally as an example of ‘best practice’ for the involvement of ‘indigenous’ African populations ^69^, this project, as well as its inconsiderate presentation of research results, was perceived as deeply offensive by the Namibian and South African San Councils and led to the development of the San Code for Ethics referenced earlier.

Because of this and other experiences, the framework requires researchers to be mindful of whether and how groups are identified in genomics research, and how research results are reported. Importantly, the framework suggests that community engagement may be one way to alleviate the potential for stigma. Genuine intellectual leadership by senior African researchers and their meaningful involvement in the preparation of manuscripts is equally important to ensure the respectful engagement with African populations and the responsible reporting of study findings.

In terms of **benefit sharing**, the framework proposes that genomics research and biobanking may bring intangible benefits in the form of general study results, social recognition, knowledge production and translation of relevant knowledge to healthcare practice. Whilst there may be some tangible benefits emanating from genomics research and biobanking in the form of (patentable) innovations or technologies, these are rare and should not be the focus of benefit sharing discussions. The framework describes, first, that it is imperative that researchers ensure that intangible benefits accrue to researchers and communities and they should be aware of this. It also describes that researchers should be mindful not to raise unrealistic expectations, and to clearly describe to communities and individuals the nature of potential benefits they can expect and those that they cannot.

**Capacity building** for African scientists is one of the central elements of the framework, and has been identified as one of the primary benefits emanating out of ongoing research endeavours such as H3Africa ^25, 70^. Building a critical mass of scholars in genomics and biobanking is essential to ensure the sustainability of these research approaches in Africa. Similarly, such a critical mass is needed to ensure that this research can be conducted by African research teams and under African intellectual leadership in the future, provided that capacity is built along the entire academic hierarchy and includes junior scientists as well as more senior ones. Importantly, capacity building would need to focus not just on training in genomics science and bioinformatics, but also in grants administration, contract negotiation, ethics and in transferable skills such as grant writing which enables sustainability of the genomics research. The expectation is that broad capacity building would ensure that research is responsive to the health needs of Africans, is sensitive to African ethical, legal and social issues, and that there is a strong avenue for the implementation of relevant research findings into national health policy and clinical practice.

With regard to the **export of samples** to other countries, the framework acknowledges that this is often viewed as problematic by research ethics committees and other regulators, not in the least because it is viewed as perpetuating inequality. For this reason, the framework proposes that export should only be permitted where researchers can outline how their work will contribute to reducing global health inequality and what measures they have put in place to strengthen the research system in the country where the samples were collected. One example would be where junior and senior African students and researchers are meaningfully involved in all aspects of the research process, including aspects that happen in non-African laboratories and universities. Material Transfer Agreements (MTAs) are fundamental to underpin the fair export of samples. While the challenges around implementing MTAs remain, guidance offered for instance by the US Veterans Health Administration ^71^ and some model MTA templates such as the Uniform Biological Materials Transfer Agreement and its related templates ^72^, could be useful in supporting institutions to navigate these challenges, arriving at MTAs that are more agreeable to collaborators in genomics research.

Whilst the framework offers some guidance on the **feedback of individual genetic research results**, it mainly proposes a range of questions that need to be considered in determining whether and under which conditions the return of research results may be appropriate in the African research context. Given the complexity of the issues, the H3Africa Consortium is developing a policy guiding researchers in how to decide which results to feedback, which expands on the summary guidance given in the framework.

Lastly, implementing a **good governance** regime is recommended in order to tie all these elements together towards optimal protection of participant in genomics research and biobanking. This should be a mechanism that provides oversight for re-use of samples and data sharing in line with the principles and elements set out in the framework. Such oversight is expected to among other things, ensure that decisions to provide access to sample and data for secondary use are sensitive to the need to promote genomics scholarship from African scientists and facilitate preferential use of data and access to samples for such scientists for a reasonable period of time. Such a preferential use provision is expected to further support African scientists, who may have challenges in engaging with the data and specimen available as fast as their counterparts due to systemic challenges such as poor power supply, poor access to academic databases for research, poor access to fast and reliable internet and so forth; or infrastructural challenges such as the availability of databases with comparable security protections that will allow for sharing data across countries and continents. In making these decisions however, it is important to find an appropriate balance between over-protection, which may hinder good science with potential benefit to humanity derivable from it. Typically, such a governance regime is achieved through the establishment of Sample Access Committees and Data Access Committee ^73^. However, each country that seeks to use the framework, as a guide may have to develop a structure that works best considering local peculiarities.

## Implementation mechanisms and amendments

The next step in our quest to consolidate and harmonize standards for African genomics research and biobanking is to engage broadly around the framework, and to develop template guidelines for adaptation.

Firstly, the framework provides the basis for further discussions and engagement with professional organisations, regulators and ethics committees across Africa. Through our consultation processes, we have built up a rich network of contacts with regulators and ethics committees at local, regional and national levels across the continent, and we are liaising with all of these to create awareness of the minimal standards described in the Framework. We are also liaising with professional science organisations including for instance the African Academy of Sciences (AAS), to explore how that organisation can take a leadership role in advancing ethical standards of genomics research and biobanking on the continent.

In terms of ensuring the incorporation of the standards outlined in the Framework into research practice, we are preparing more detailed guidelines that expand on the items in the Framework. This resource will be publicly available for use by ethics committees across the continent, and the hope is that ethics committees and national ethics councils will adapt and adopt the guidelines so that they become the gold standard for national regulation of genomics and biobanking. In order to ensure that they do, we will continue our engagement activities with committees and national councils, which are usually in the form of side meetings at the annual meetings of the H3Africa Consortium. In addition, we are increasingly involved in offering training to national and local ethics committees, which are invaluable in ensuring awareness of the Framework and guidelines emanating from them. Although there are no plans to commission formal evaluations on the uptake of the Framework, the H3Africa Working Group on Ethics and Community Engagement shall continue to collect information on references to the framework in publications as well as its use in country guidelines. 

## Disclaimer

The views expressed in this article are those of the authors. Publication in *AAS Open Research* does not imply endorsement by the AAS.

## Data availability

No data are associated with this article.
