# Supplementary material for: Model framework for governance of genomic research and biobanking in Africa – a content description
Source: AAS Open Res. 2018 Dec 12;1:13. Originally published 2018 Apr 18. [Version 2] doi: 10.12688/aasopenres.12844.2 (PMC6354903; doi:10.12688/aasopenres.12844.2)
Supplement: Peer review history

# Peer review: Reviewer response for version 1

**DOI:** 10.21956/aasopenres.13910.r26458

**Published:** 2018-06-26

**Authors:** Don Chalmers

**Affiliations:** 1 Centre for Law and Genetics, Faculty of Law, University of Tasmania, Hobart, Tas, Australia

This is a very well written and valuable account of the development of an ethical governance framework for genomic research and biobanking in Africa.

 I strongly recommend indexing for the following reasons

  

1. The article sets a clear context for the H3Africa and the development of an autochthonous African model of consent for sharing genomic data and samples which respects autonomy; consent; ownership of samples and data; privacy and confidentiality; and, benefit sharing.
2. The article makes a valuable contribution to the continuing development of an overarching African philosophical basis for a “.. framework …[with]a more generic communal or solidarity-based worldview”. This is different, as is the focus on the “..central importance on reciprocity, consultation and accountability’. This draws a clear distinction between the proposed African model and conventional western participant-focused ethical approaches, which can accompany “parachute research”, mentioned in the Introduction. 
3. The article details the elements of this African best-practice genomics and biobanking framework based on “African intellectual leadership; Consent; Community engagement; Ethics review; Avoidance of group harm and stigma; Benefit sharing; Capacity building; International collaboration and export of samples; Feedback of individual genetic findings; and Good governance.
4. The implementation agenda for the H3Africa framework is briefly set out. Specifically noted is future engagement with professional organisations, regulators and ethics committees before drafting more detailed guidelines and engagement activities with ethics committees and national councils.

 

 I have some suggestions for the authors

  

1. The brief mention of the principle of “ubuntu” is interesting and is there any more recent discussion since the footnote 35 2007 reference?
2. In the section on “Core Principles and elements’, the authors mention ‘five core principles” but only list four (a to d). They need to add the fifth
3. The authors highlight the elements of their proposed framework (see 3 above) but do not include any discussion on the principle of “African intellectual leadership”.
4. There has been a working connection between the H3Africa and the GA4GH and a reference could be included to this at: https://www.ga4gh.org/news/ffY_2As3q-iv39gB2Un4Bw.article. 
5. In the acknowledgements, the authors list the key resources of the Global Alliance for Genomics and Health (GA4GH) *Framework for the Responsible Sharing of Genomic and Health-Related Data*; the OECD *Guidelines on Human Biobanks and Genetic Research Databases*, 2009 the Wellcome Trust *Framework on the* *feedback of health-related findings in research*, 2014 the European Commission’s reports on *Global Governance of Science*, 2009 and *Ethical and Regulatory Challenges to Science and Research Policy at the Global Leve*l 2012 and the 22 African country-specific ethics guidelines.
  These global collaborations and interactions are worthy of reference and comment in the text at the section “Key elements of the Framework for Best Practice’. This records that the authors are striking out on a path for Africa but one informed by international developments and collaborations.

 

 Declaration of interest I have met Littler K and De Vries J but have never published with either.

I confirm that I have read this submission and believe that I have an appropriate level of expertise to confirm that it is of an acceptable scientific standard.

# Response

**Published:** 2018-11-12

**Authors:** aminu yakubu

Dear Prof Chalmers,

 Thank you for reviewing our paper. The team greatly valued comments and recommendations you provided. We have made necessary changes to the paper (which we shall be submitting shortly) in response to your comments. We have considered the suggestion for indexing and are in discussion with the leadership of the Consortium on this regard. Here is a summary of our responses to your specific comments on the paper:

 I have some suggestions for the authors

 The brief mention of the principle of “ubuntu” is interesting and is there any more recent discussion since the footnote 35 2007 reference?

- Thank you. Though our aim was not to dwell much on describing Ubuntu, we have however added a little more text and references in response to this comment. We feel that provides the additional information suggested without going too deep.

 

 In the section on “Core Principles and elements’, the authors mention ‘five core principles” but only list four (a to d). They need to add the fifth

- This was one oversight; we actually have only four principles. Error corrected, thank you.

The authors highlight the elements of their proposed framework (see 3 above) but do not include any discussion on the principle of “African intellectual leadership”.

- We have now included some more discussion on this as suggested. We have linked this issue with the 10/90 gap and the issues raised about poor diversity and inclusion in genomics research. Thank you.

There has been a working connection between the H3Africa and the GA4GH and a reference could be included.

- Thank you for this observation. We believe the acknowledgement and the new citation (in response to comment 5 below) is sufficient to demonstrate the connection between the two for the purposes of this paper.

 

 In the acknowledgements, the authors list the key resources of the Global Alliance for Genomics and Health (GA4GH) *Framework for the Responsible Sharing of Genomic and Health-Related Data*; the OECD *Guidelines on Human Biobanks and Genetic Research Databases*, 2009 the Wellcome Trust *Framework on the* *feedback of health-related findings in research*, 2014 the European Commission’s reports on *Global Governance of Science*, 2009 and *Ethical and Regulatory Challenges to Science and Research Policy at the Global Leve*l 2012 and the 22 African country-specific ethics guidelines.

 These global collaborations and interactions are worthy of reference and comment in the text at the section “Key elements of the Framework for Best Practice’. This records that the authors are striking out on a path for Africa but one informed by international developments and collaborations.

- Thank you. We have included some reference while introducing the “Key Elements” section.

# Peer review: Reviewer response for version 1

**DOI:** 10.21956/aasopenres.13910.r26476

**Published:** 2018-06-25

**Authors:** Ellen Wright Clayton

**Affiliations:** 1 Center for Biomedical Ethics and Society, Vanderbilt University Medical Center (VUMC), Nashville, TN, USA

This is an important paper setting forth guiding principles for genomics research in Africa.  The document is clear and well written and will be quite helpful for the work of putting concrete mechanisms in place that protect the interests of individuals and communities while building local capacity and advancing research to improve health. 

 I have only one substantive comment, which is that it would be helpful to say more about what the authors mean by community engagement, specifying to what extent and on what topics the community should have input.  This is often a contested issue.

 My other comments are minor:

 Under core principles, there is an extra "of" on the second line.

 I saw only four principles, not five.

 On line 9 under consent process, I assume that "it" refers to broad consent.  If so, that should be stated explicitly.

 Under avoidance of group harm, there should not be a comma after note 52.

I confirm that I have read this submission and believe that I have an appropriate level of expertise to confirm that it is of an acceptable scientific standard.

# Response

**Published:** 2018-11-12

**Authors:** aminu yakubu

Dear Prof Clayton

Thank you for reviewing our paper. We are pleased to learn that you consider it an important contribution to genomics research governance. We carefully reviewed your comments and taken the necessary actions and/or made the necessary modifications. Please find a summary below:

This is an important paper setting forth guiding principles for genomics research in Africa.  The document is clear and well written and will be quite helpful for the work of putting concrete mechanisms in place that protect the interests of individuals and communities while building local capacity and advancing research to improve health. 

I have only one substantive comment, which is that it would be helpful to say more about what the authors mean by community engagement, specifying to what extent and on what topics the community should have input.  This is often a contested issue.

- Thank you for this comment. Indeed we share in your perspective but wanted to strike a balance between presenting CE within the scope of this descriptive paper and going in-depth to educate our readers more on the topic. However, because of this observation, we have added a bit more context.

My other comments are minor:

 

- Under core principles, there is an extra "of" on the second line.
  - Line removed, thank you.
- I saw only four principles, not five.
  - This was an oversight; there are actually four. This has been corrected.
- On line 9 under consent process, I assume that "it" refers to broad consent.  If so, that should be stated explicitly.
  - Yes, it does. We have added that specificity. Thank you.
- Under avoidance of group harm, there should not be a comma after note 52.
  - Addressed, thank you.

# Peer review: Reviewer response for version 1

**DOI:** 10.21956/aasopenres.13910.r26442

**Published:** 2018-06-21

**Authors:** Kieran O'Doherty, Kim Chuong

**Affiliations:** 1 Department of Psychology, University of Guelph, Guelph, ON, Canada

This article describes an ethics framework for genomics research and biobanking in Africa, which is valuable in the African public health context and to address global health inequality. The article is well-written and concise in its summary of the core principles and key elements of the framework. We see the contribution of the framework in the context of ELS implications of genomics and biobanks in its emphasis on the need for research to be sensitive and respectful of diverse cultures, values and belief systems of African populations. Also of significance is the goal to enhance capacity building and genuine intellectual participation of African researchers and stakeholders in genomics research. The authors of this article are to be commended for this important work.

 Overall, the manuscript will make an important contribution. We have only minor suggestions for improvement. Although the key elements of the framework are well-presented, the authors do not address potential challenges that may be encountered while applying those key elements. We realise that the purpose of the article is to lay out the framework, and so does not address implementation issues. However, some additional detail may be good to add, in particular in the context of community engagement, which the authors emphasize as a key component for ensuring best practice in genomics research and biobanking in Africa. However, it is not clear from the framework what the authors mean by “genuine community engagement”. There is a rich literature on engaging community members in research and the values and challenges of doing so. Community engagement goes beyond the meaningful involvement of African researchers to include individuals from the broader population and specific groups, and will need to address issues such as power dynamics between researchers who are considered experts and community members.

Other issues:

 On p. 3. The authors mention that “The process of engagement that led to the development of this framework is reported in another manuscript.” A reference to this manuscript should be provided.

 The paragraph on export of samples to other countries is incomplete (p. 6). 

 The “implementation mechanisms and amendments” section (p. 7) describes the next steps for raising awareness and engaging health organisations and national councils with the framework. It would be great to have more detail regarding future evaluation of the framework and the Working Group’s engagement activities.

We confirm that we have read this submission and believe that we have an appropriate level of expertise to confirm that it is of an acceptable scientific standard.

# Response

**Published:** 2018-11-20

**Authors:** aminu yakubu

Dear Drs O'Doherty and Chuong,

 On behalf of my co-authors, I wish to thank you for taking the time to review our paper. We thought your comments were insightful and have made some changes to the paper in response. Please find below a summary of our responses to your comments. We shall submit the revised version of the paper shortly.

 Overall, the manuscript will make an important contribution. We have only minor suggestions for improvement. Although the key elements of the framework are well-presented, the authors do not address potential challenges that may be encountered while applying those key elements. We realise that the purpose of the article is to lay out the framework, and so does not address implementation issues. However, some additional detail may be good to add, in particular in the context of community engagement, which the authors emphasize as a key component for ensuring best practice in genomics research and biobanking in Africa. However, it is not clear from the framework what the authors mean by “genuine community engagement”. There is a rich literature on engaging community members in research and the values and challenges of doing so. Community engagement goes beyond the meaningful involvement of African researchers to include individuals from the broader population and specific groups, and will need to address issues such as power dynamics between researchers who are considered experts and community members.

- Thank you for this comment. Indeed we share in your perspective but wanted to strike a balance between presenting CE within the scope of this descriptive paper and going in-depth to educate our readers more on the topic. However, because of this observation, we have added a bit more context and specify what we mean by genuine (vs tokenistic) engagement.

Other issues:

 On p. 3. The authors mention that “The process of engagement that led to the development of this framework is reported in another manuscript.” A reference to this manuscript should be provided.

- Thank you. We have cited the paper, which is now “in press” with the BMC Medical Ethics journal 

 

 The paragraph on export of samples to other countries is incomplete (p. 6). 

- This omission is regretted. We wanted to make reference to the UBMTA and the VA guideline on MTAs. We have now done so. Thank you.

 

 The “implementation mechanisms and amendments” section (p. 7) describes the next steps for raising awareness and engaging health organisations and national councils with the framework. It would be great to have more detail regarding future evaluation of the framework and the Working Group’s engagement activities.

- Thank you for this comment. We have added to our description of future steps.
